# Cochlear Implantation: Long-Term Effect of Early Activation on Electrode Impedance

**DOI:** 10.3390/jcm13113299

**Published:** 2024-06-03

**Authors:** Asma Alahmadi, Yassin Abdelsamad, Medhat Yousef, Fida Almuhawas, Ahmed Hafez, Farid Alzhrani, Abdulrahman Hagr

**Affiliations:** 1 King Abdullah Ear Specialist Center (KAESC), King Saud Medical City, King Saud University, Riyadh 11411, Saudi Arabia; myousef@ksu.edu.sa (M.Y.); fmuhawas@ksu.edu.sa (F.A.); faalzhrani@ksu.edu.sa (F.A.); hagr@ksu.edu.sa (A.H.); 2 Research Department, MED-EL GmbH, Riyadh 11563, Saudi Arabia; yassin.samad@medel.com (Y.A.); ahmed.hafez@medel.com (A.H.); 3 Audio Vestibular Unit, ENT Department, Menoufia University, Menoufia 32928, Egypt

**Keywords:** green cochlea, cochlear implant, sustainability, electrode impedance, early activation, classical activation, post-op outcomes

## Abstract

**Objectives**: The growing adoption of cochlear implants (CIs) necessitates understanding the factors influencing long-term performance and improved outcomes. This work investigated the long-term effect of early activation of CIs on electrode impedance in a large sample of CI users at different time points. **Methods**: A retrospective study on 915 ears from CI patients who were implanted between 2015 and 2020. According to their CI audio processor activation time, the patients were categorized into early activation (activated 1 day after surgery, n = 481) and classical activation (activated 4 weeks after surgery, n = 434) groups. Then, the impact of the activation times on the electrode impedance values, along the electrode array contacts, at different time points up to two years was studied and analyzed. **Results**: The early activation group demonstrated lower impedance values across all the electrode array sections compared to the classical activation at 1 month, 1 year, and 2 years post-implantation. At 1 month, early activation was associated with a reduction of 0.34 kΩ, 0.46 kΩ, and 0.37 kΩ in the apical, middle, and basal sections, respectively. These differences persisted at subsequent intervals. **Conclusions**: Early activation leads to sustained reductions in the electrode impedance compared to classical activation (CA), suggesting that earlier activation might positively affect long-term CI outcomes.

## 1. Introduction

The most effective sensory prosthesis in the world, cochlear implants (CIs), have been the focus of extensive study and development in recent years [1]. This has primarily been driven by broadening the candidacy criteria to include people of all ages and encompasses indications like single-sided deafness and ski-slope hearing loss [2,3,4]. Despite the technological advances in cochlear implantation, inserting the array into the cochlea is nonetheless still an insertion of a foreign body into a biologically sensitive environment. This presence elicits a series of biological responses, leading to the formation of a fibrotic capsule surrounding the implant, which is a major contributor to an increase in a fundamental electrical property in CI, named electrode impedance [5,6,7].

Electrode impedance reflects the resistance of the electrode array channels encountered in the cochlear environment. Measuring this impedance is a standard practice in all manufacturing companies as part of determining the functionality and integrity of the implants [8]. Calculating the impedance typically involves sending a small current through each channel and measuring the voltage response. The impedance calculations are based on Ohm’s law, with the software automatically processing voltage and current data to determine the impedance values, considering both the resistive and reactive components [9]. In routine practice, impedance changes are monitored over time, as they are an indicator of the electrode-tissue interface and cochlear environment, which is vital for the long-term performance of the implant [10]. Significant changes in the impedance values can signal issues such as electrode degradation, formation of fibrous tissue surrounding the electrode, or modifications to the cochlea’s fluid environment [10,11]. Furthermore, the impedance data of fibrous tissue and new bone growth in the cochlea resulting from electrode array implantation influences residual hearing [12].

The association between the formation of fibrotic capsules and the increase in impedance is of particular interest. Previous reports showed that impedance significantly increases in correlation with the extent of the fibrotic sheath around the array [13]. Research demonstrates that following implantation, the impedance rises for a few weeks before beginning to decrease and stabilize upon device activation, probably due to the electrical stimulation that distorts the adherence of the fibrous tissue to the electrode [14]. Increased impedance in the first few weeks after implantation can lead to higher voltages needed to provide the necessary stimulation current, resulting in increased energy consumption and shorter durability of the CI audio processor batteries [10]. Previously, corticosteroids were proposed to slow the development of fibrotic tissue due to their anti-inflammatory properties [15]. However, it was found that the response to corticosteroids was variable across the CI recipients [10]. Recently, it was shown that steroid-eluting electrodes are safe for use in humans and show low impedances [16]. However, to date, no cochlear implants with drug-eluting electrodes are market-approved. Furthermore, the additional cost associated with corticosteroids as part of the newer formulations in cochlear implants is unknown. Therefore, to save costs, this analysis sheds light on the beneficial potential of early activation.

Classically, CIs are activated approximately four weeks after implantation to avoid wound disruption [17]. On the other hand, new data indicate that early activation (EA) of CIs is safe, practical, and cost-effective [18,19,20,21,22,23]. Furthermore, authors have recently hypothesized that early activation can be significantly associated with a decreased electrode impedance one month after implantation compared to classical activation [11]. However, such a hypothesis has seldom been studied, and the small sample size limits the current published literature. Given the current gap in the published literature, the goal of the current investigation was to determine how early activation versus classical activation (CA) affected the electrode impedance variations over time in CI recipients/users. The CI activation could be routinely performed on the first day after implantation if there are no contraindications, e.g., dizziness, skin irritation, pain, or discomfort before or after activation [24]. At our center, the long experience and large sample size of CI recipients enable us to study the early electrical effect of the CI fitting parameters and outcomes. The primary aim of the present study, therefore, was to investigate the long-term effect of early electric stimulation on the electrode impedance. The secondary objective was to study the influence of different activation times on the electrode impedance over time.

## 2. Materials and Methods

This retrospective cohort study followed the Declaration of Helsinki guidelines and was approved by the local institutional review board (E-21-5737) on 16 February 2021.

### 2.1. Participants

To be included in this study, potential participants had to have: (1) received a CI unilaterally or bilaterally with a lateral wall electrode array (MED-EL, Innsbruck, Austria) via the round window at our tertiary CI center between 2015 and 2020; (2) normal inner ear anatomy; (3) a full CI array insertion; (4) normal intraoperative measurements; (5) a normal array position within the cochlea, as confirmed via mastoid radiography; (6) had a postoperative follow up for at least 2 years; and (7) had an electrode array that covers at least 1.5 to 2 turns of the cochlea. Potential participants were excluded if they (1) had a history of meningitis or head trauma, (2) were CI reimplantation cases, (3) had been on corticosteroids for an extended period for reasons unrelated to the surgical operation, (4) had a device failure, or (5) showed an electrode migration.

Upon study inclusion, the participants were divided into 2 groups based on how soon after CI provision their audio processor was activated. The study group, designated as early activation (EA), had their audio processor activated 1 day after surgery, as per our clinical routine since 2015. The control group, designed as classical activation (CA), had their audio processor activated 4 weeks after surgery, which is a general worldwide norm with CI recipients and was sometimes routinely performed in our clinic in the past and still is in some cases according to the surgeon’s preferences.

#### Surgical Procedure and Electrode Impedance Measurements

The CI implantation surgery and electrode impedance measurements were conducted with the same steps for all the participants. The CI implantation surgery was conducted under general anesthesia. A post-auricular incision facilitated access to the mastoid bone, followed by mastoidectomy and posterior tympanotomy to expose the round window niche, with careful preservation of the facial nerve and other critical structures. We followed a minimal incision technique [24] at our clinic for many reasons, such as the shorter period of wound healing, dismissing the need for shaving, and dispensing with the need for dressing after the surgery. The electrode array was inserted via the round window. The emphasis was on achieving a smooth and complete insertion into the cochlea, minimizing potential trauma to the cochlear structures. Intraoperative measurements, including impedance testing to ensure the implant’s functionality and integrity, and the evoked compound action potential were performed to confirm the nerve response. The surgical site was closed in layers with very close sutures. After implantation, mastoid radiography was performed to verify the correct electrode array placement within the cochlea.

For the EA group, the electrode impedance was measured and analyzed at 4 intervals: just before activation and at 1 month, 1 year, and 2 years post-implantation. The CA group was measured just before activation (at 1 month after implantation) and at 1- and 2-years post-implantation. The MAESTRO 9.0 fitting software (MED-EL) was used to perform the impedance measurements. The electrode array that the participants were implanted with has 12 electrode contacts, which are distributed from the most apical (electrode 1) to the most basal (electrode 12). During the analysis, the electrode array was divided into three sections: apical (electrodes 1–4), middle (electrodes 5–8), and basal (electrodes 9–12).

### 2.2. Sample Size

A total sample size of 100 ears for each activation group was sufficient to achieve 80% power for the study at a 5% level of significance, considering the effect size in terms of the electrode impedance between the 2 groups at 1-month post-activation (Cohen’s d = 0.43) from Alhabib et al.’s 2021 study [8], after adding a 15% loss of follow-up rate.

### 2.3. Statistical Analysis

Version 4.2.2 of the R software was used for the statistical analysis. The interquartile range (IQR) and median were used to describe the continuous data, while the categorical data were summarized using the count and percentage. The electrode contacts that showed high impedance were excluded and treated as missing, while the other functioning electrode contacts from the same patients were included and analyzed. The Shapiro–Wilk test was used to verify the normality assumptions, and the multiple pairwise Bonferroni-adjusted Wilcoxon signed-rank test was employed to evaluate the differences in electrical impedance within the research groups between various time points. Generalized Estimating Equation (GEE) models were employed to assess the marginal effects of early activation on electrical impedance over time. The model is an extension of the generalized linear model that considers both the non-normally distributed nature of the impedance values as well as the longitudinal nature of the data (for repeated measures at different time points). The GEE model showed the impact of the interaction between the time since implantation and the type of activation (early or classical) at the different electrode sections (apical, middle, basal) individually. The results of the GEE model were reported as the β coefficient and its 95% confidence interval for the EA in comparison to the CA groups. Significance was set to a *p*-value ≤ 0.05.

## 3. Results

### 3.1. Participants

The inclusion/exclusion criteria were met in 915 ears, with 481 in the EA group (245 right ear, 236 left ear) and 434 in the CA group (256 right ear, 178 left ear). All the participants received a MED-EL CI. No participants experienced dizziness, skin irritation, pain, or discomfort before or after activation. The patients’ baseline demographics, including age at implantation in years as well as gender, are represented in Table 1 for the early and classical activation groups.

### 3.2. Electrode Impedance Values

**Figure 1:**
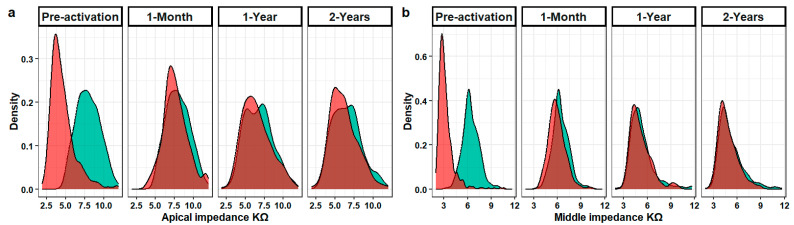

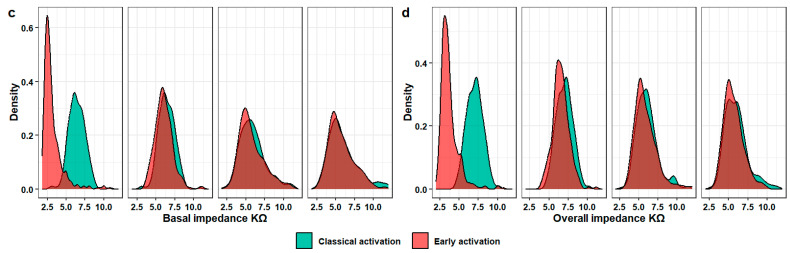
Shows the density plots for each research group’s electrode impedance at various electrode sections and time intervals. The following examples illustrate the skewed distributions: (**a**) the apical electrode impedance values at different times in both groups, (**b**) the middle electrode impedance values at different times in both groups, (**c**) the basal electrode impedance values at different times in both groups and (**d**) the overall electrode impedance values at different times in both groups.

**Figure 2:**
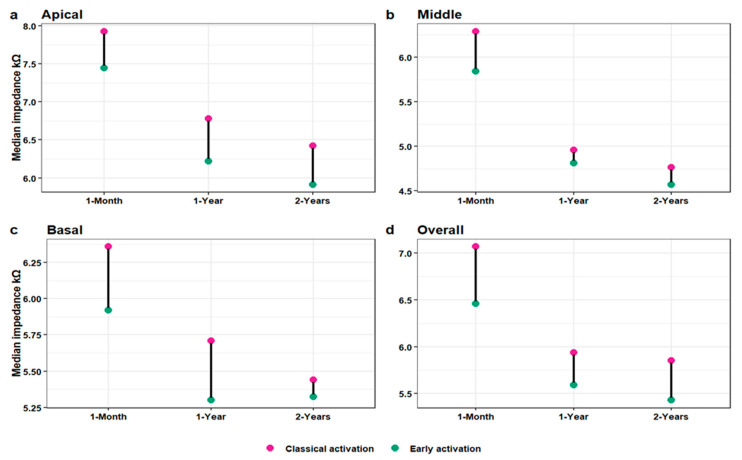
Dumbbell chart of the difference in the median electrode impedance between the study groups at each electrode section at each interval post-implantation. Each pair of points adjacent to the same time point shows the corresponding medians of both groups and is connected by a black horizontal line representing the difference: (**a**) for the apical electrode section, (**b**) for the middle electrode section, (**c**) for the basal electrode section, and (**d**) for the overall electrode.

The EA group’s median impedance values were lower than those of the CA group at each electrode section at all the intervals post-implantation (*p* values < 0.001 for all). The skewness of all the impedance values for each electrode section at each interval in both study groups was detected by the density plots shown in Figure 1. Within the group, the values decreased at each interval for each electrode section except for the EA group’s basal values between the 1- and 2-year intervals (Figure 2). The groups’ impedance values for each electrode section at each interval are reported in Table 2.

### 3.3. The Way the Study Groups Interacted at Various Times

#### 3.3.1. The Apical Electrodes

One month post-op, the EA group exhibited a statistically significant reduction in the apical electrode impedance of approximately 0.34 kΩ compared to the CA group. The trend persisted at the 1-year and 2-year post-op intervals, where the EA group demonstrated sustained reductions in the electrode impedance of approximately 0.2 kΩ and 0.29 kΩ, respectively, relative to the CA group. The β coefficients for the EA group, along with their interaction with the time point, were consistently significant across all the intervals (Table 3).

#### 3.3.2. The Middle Electrodes

One month after implantation, the middle electrode impedance in the EA group experienced a notable and significant reduction of approximately 0.46 kΩ compared to the CA group. Moving on to the 1-year post-op interval, the EA group also exhibited a significant decrease in the electrode impedance of approximately 0.19 kΩ relative to the CA group. Similarly, at the 2-year post-op, a significant decrease of about 0.34 kΩ in the middle electrode impedance was observed in the EA in comparison to the CA group. The β coefficients for the EA group and their interaction with the time point remained consistently significant across all the time intervals (Table 3).

#### 3.3.3. The Basal Electrodes

One month after implantation, a notable decrease in the basal electrode impedance was evident in the EA group, with a reduction of approximately 0.37 kΩ compared to the CA group. Moving on to the 1-year post-op period, the EA group demonstrated a significant decrease in the electrode impedance, declining by approximately 0.11 kΩ relative to the CA group. Similarly, at the 2-year post-op, the basal electrode impedance exhibited a significant decrease of about 0.2 kΩ in the EA compared to the CA. The β coefficients for the EA group and their interaction with the time point consistently retained significance across all the intervals (Table 3).

#### 3.3.4. The Overall Impedance

One month post-op, a statistically significant decrease was observed in the overall electrode impedance (calculated as the average of the 12 electrodes) of the EA group, reducing by approximately 0.47 kΩ compared to the CA group. Shifting to the 1-year post-op timeframe, the overall electrode impedance exhibited a significant decrease in the EA group, diminishing by about 0.17 kΩ relative to the CA group. Furthermore, at the 2-year post-op interval, the overall electrode impedance had significantly decreased in the EA group, showing a reduction of about 0.29 kΩ compared to the CA group. The β coefficients for the EA group, along with their interaction with the time point, consistently displayed significance across all the time intervals (Table 3).

### 3.4. Changes in Electrode Impedance over Time for Each Group

**Figure 3:**
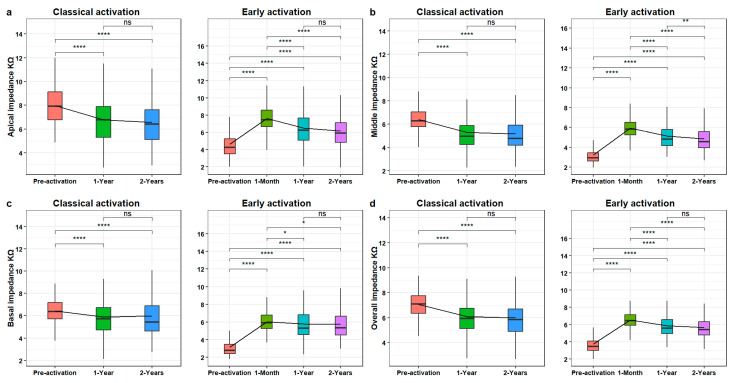
Boxplots showing the electrode impedance variations at different electrode sections over time after implantation within the EA and CA groups: (**a**) for the apical electrode section, (**b**) for the middle electrode section, (**c**) for the basal electrode section, and (**d**) for the overall electrode. ns = non-significant, *p* ≤ 0.05 is indicated *, *p* ≤ 0.01 by **, and *p* ≤ 0.0001 by ****.

As Figure 3 shows, for the EA group, the impedance values significantly increased from pre-activation to 1-month post-implantation and then significantly decreased from 1 month to 1 year. For all the sections except the middle, no significant difference was found between 1 year to 2 years.

For the CA group, for each electrode section, a significant decrease was found between pre-activation and both 1- and 2-years post-implantation. No significant difference was found between 1 year and 2 years. A detailed comparison of each mode of activation at the different time points is presented in Table 4.

## 4. Discussion

The findings of our study showed that, when compared to conventional activation (CA), early activation (EA) of Cis consistently and significantly lowers the overall electrode impedance values during a two-year post-op period. Further analysis of the different electrode sections—apical, middle, and basal—also showed distinct patterns of impedance reduction, with the EA group showing substantial reductions. However, the reduction of the electrode impedance with EA was prominent in the apical section, suggesting a more powerful and section-specific effect of EA on the impedance measures.

The current body of evidence shows a positive correlation between the electrode impedance values and the formation of fibrotic sheaths around the CI electrode array [25]. Increased electrode impedance values can lead to higher voltages needed to provide the necessary stimulation current and shorten the durability of the CI audio processor batteries [10]. High electrode impedance indicates that to sustain the given charge, the implant needs to produce a greater voltage. This has two negative effects: first, it quickly depletes the device’s charge; second, and more significantly, it disperses the current spread across more SGN, which lowers the frequency resolution and, consequently, the perceived sound quality. Therefore, the patient’s performance is often better when their electrode impedance is low [25].

In the present study, we found that the early electric stimulation during EA led to sustained reductions in the electrode impedance over 2 years after implantation. In comparison to the CA group, the electrode impedance values in the EA group were consistently lower. Such findings were different from other previous reports such as that by Alhabib et al. [11] on 80 ears, which showed that EA led to lower electrode impedance values compared to CA in the first month after implantation and nothing onward; and also, as by Saoji et al. [26] on 51 patients, who reported no statistically significant differences in the impedances among the different activation groups. This could be attributed to the big difference between the current work and these studies in the sample size, follow-up duration, and/or different electrodes. Although the exact mechanisms underpinning the beneficial effects of EA on the electrode impedance are unclear, several explanations can be hypothesized based on the positive impacts of electricity on human health [27].

One potential explanation is the anti-inflammatory effect of early electrical stimulation, which alters the surface charge of the cochlear implant, reducing protein adsorption to its surface, and preventing the proliferation of inflammatory cells also activates anti-inflammatory markers, modulating the immune response and reducing inflammatory reactions, thus limiting fibrosis around the electrode array [28]. This could result in maintaining lower electrode impedance levels by minimizing tissue reactions known to increase impedance. Additionally, the anti-proliferative actions of electrical stimulation may prevent fibrous tissue from growing, a typical response to the foreign body of the CI, helping to preserve a more favorable electrode-tissue interface [29]. Another aspect to consider is the enhanced reinnervation that early stimulation might promote, creating an environment conducive to the regrowth or maintenance of nerve fibers near the electrodes [30]. This could lead to a more efficient signal transmission, reflected in the lower electrode impedance values. Furthermore, the direct benefits of nerve stimulation, provided consistently through EA, may prevent nerve fiber degeneration and support their functional integrity [31], thus contributing to lower electrode impedance values by maintaining an optimal nerve–electrode interface. These mechanisms suggest that EA following cochlear implantation plays a crucial role in optimizing the implant’s performance and longevity by maintaining a healthier electrode-tissue interface. One of the other options to limit the growth of fibrotic tissue and decrease the electrode impedance is corticosteroids because they have anti-inflammatory qualities. However, there are currently no authorized Cis on the market that use drug-eluting electrodes. Moreover, it is unclear how much more corticosteroids, which are a component of more recent cochlear implant formulations, will cost [10,15].

Our study’s results revealed varied electrode impedance patterns across different electrode sections, particularly the notable reductions in the apical section among the EA group, underscoring the section-specific influence of EA on the electrode impedance outcomes. This might suggest that different areas of the cochlea react differently to electrical stimulation from the CI. The apical section is responsible for processing lower frequencies, where most residual hearing exists, and the field of hearing preservation. This area may have unique anatomical and physiological characteristics that render it more susceptible to the benefits of early electrical stimulation, such as reduced inflammatory and fibrotic responses [12]. This differential tissue reaction could be another factor that led to the observed lower impact of EA on the apical electrodes. Furthermore, the variance in the electrical stimulation thresholds and efficiency along the length of the cochlea might contribute to these regional disparities. These findings not only have significant implications for the tailoring of CI programming, ensuring that strategies are adapted to the specific responses of different cochlear sections, but also highlight the necessity for further research. Understanding these regional-specific effects is crucial for advancing CI design and activation methodologies, potentially leading to more individualized and effective hearing solutions.

According to our research, the apical section of the CI electrode array often has greater electrode impedance values than the basal section, which is in line with many studies that reported similar trends. Research has demonstrated that the electrode position has a systematic effect on the impedance values, with greater values corresponding to closer proximity to the cochlear wall and higher impedances in contacts put more apically [11]. For instance, one study reported that the highest impedances occurred on apical contacts, with the values decreasing toward the middle and basal contacts [32]. Additionally, another study found that the impedance values were higher for apical electrodes compared to basal electrodes, with the average impedance decreasing from the apical to the basal electrode positions [33]. As a result, it is shown that the apical region of the electrode array typically has higher impedance values, which reflects the impact of the electrode position on impedance measurements.

As explained, the differences in the electrode impedance values are statistically significant between both groups, but they are small values except for the 1-month point. Saoji et al. in 2022 [26] suggested that differences in electrode impedance <1 kiloohm may not be clinically significant. So, while in the short-term EA reduces the impedances quite relevantly, in the long run, the differences in the current study are only a few hundred Ohms. However, this might still be useful in patients with a thick skin flap or can reduce the cumulative energy costs over the years.

Only a few research studies, as far as we know, examined electrode impedance changes over the course of two years following implantation. The strengths of this study include the large sample size (a cohort of 915 ears) and the long-term follow-up among different activation modes; however, we acknowledge the existence of certain limitations. The retrospective nature of the study might have led to unmeasured variables influencing the electrode impedance, such as variations in the surgical techniques, electrodes, patient adherence to post-op care, and the implication of post-operative performance.

This work suggests that the early activation of a CI could help increase the battery life due to decreasing the electrode impedance values. It has been reported that the higher electrode impedance quickly depletes the device’s charge and shortens the batteries’ duration [10]. Some studies reported that the increased battery life could lead to reduced resource consumption, a lower carbon footprint, a circular economy, and resource conservation [34,35]. Thus, we believe that the current endeavors to reduce the electrode impedance values in CI users might enhance the battery life and contribute to global sustainability initiatives. This could serve as a first step toward implementing eco-friendly or green cochlear implantation.

## 5. Conclusions

In conclusion, this study suggests a beneficial impact of early activation on the electrode impedance in CIs over a 2-year post-op period. The results demonstrated that EA was associated with a significant and sustained reduction in the electrode impedance, with this effect being particularly pronounced in the apical electrodes. The observed temporal changes in impedance highlight the potential of early activation strategies in maintaining lower impedance values, possibly enhancing the overall performance and durability of CIs and providing better counseling for many patients. We hope this will lead to better hearing preservation and provide more patients with better outcomes in CI treatment. However, further studies are needed to confirm our findings.

## Figures and Tables

**Table 1:** Patients’ baseline demographics among the early and classical activation groups are represented as the median (interquartile range) or count (percentage).

Side of the Ear	Demographics		Early Activation (N = 481 Ears)	Classical Activation (N = 434)	Total (N = 915)
Right-sided N = 501 (54.8)	Age at implantation (years)	Median (IQR)	4.0 (2.0 to 6.0)	3.0 (2.0 to 5.0)	3.0 (2.0 to 4.0)
		Min–Max	0–52	0–54	0–54
	Gender	Male	136 (28.3)	129 (29.7)	265 (29.0)
		Female	109 (22.7)	127 (29.3)	236 (25.8)
Left-sided N = 414 (45.2)	Age at implantation (years)	Median (IQR)	4.0 (2.0 to 5.0)	3.0 (2.0 to 4.0)	3.0 (2.0 to 4.0)
		Min–Max	0–67	0–36	0–67
	Gender	Female	112 (23.3)	85 (19.6)	197 (21.5)
		Male	124 (25.8)	93 (21.4)	217 (23.7)

**Table 2:** Descriptive analysis of the apical, middle, basal, and overall electrode impedance for each group at each interval; data are represented as the median and interquartile range—IQR.

Area of Array	Interval		Early Activation	Classical Activation
Apical	Pre-activation	N	447	227
		Median KΩ (IQR)	4.23 (3.50–5.22)	7.93 (6.78–9.12)
	1 M post-op	N	232	--
		Median KΩ (IQR)	7.45 (6.65–8.57)	--
	1 Y post-op	N	240	256
		Median KΩ (IQR)	6.22 (5.08–7.65)	6.78 (5.29–7.89)
	2 Y post-op	N	222	249
		Median KΩ (IQR)	5.91 (4.84–7.12)	6.42 (5.12–7.63)
Middle	Pre-activation	N	450	240
		Median KΩ (IQR)	2.96 (2.63–3.47)	6.29 (5.80–7.05)
	1 M post-op	N	233	--
		Median KΩ (IQR)	5.84 (5.26–6.53)	--
	1 Y post-op	N	243	261
		Median KΩ (IQR)	4.81 (4.18–5.80)	4.96 (4.24–5.88)
	2 Y post-op	N	224	250
		Median KΩ (IQR)	4.57 (3.97–5.59)	4.77 (4.18–5.92)
Basal	Pre-activation	N	450	239
		Median KΩ (IQR)	2.77 (2.40–3.46)	6.36 (5.72–7.18)
	1 M post-op	N	233	--
		Median KΩ (IQR)	5.92 (5.28–6.75)	--
	1 Y post-op	N	241	257
		Median KΩ (IQR)	5.30 (4.59–6.80)	5.71 (4.71–6.73)
	2 Y post-op	N	220	245
		Median KΩ (IQR)	5.33 (4.55–6.67)	5.44 (4.62–6.89)
Overall	Pre-activation	N	451	240
		Median KΩ (IQR)	3.44 (2.98–4.06)	7.07 (6.32–7.75)
	1 M post-op	N	233	--
		Median KΩ (IQR)	6.46 (5.90–7.12)	--
	1 Y post-op	N	243	260
		Median KΩ (IQR)	5.59 (4.94–6.57)	5.94 (5.12–6.75)
	2 Y post-op	N	224	250
		Median KΩ (IQR)	5.43 (4.80–6.34)	5.86 (4.89–6.68)

**Table 3:** Generalized Estimating Equation model results exploring the impact of early activation on the electrode impedance changes over time on different sections of the electrode array, post-operatively, compared to classical activation. *** indicates *p* ≤ 0.001.

Array	Interval	Coefficient (β)	95% Confidence Interval		*p* Value
			Lower Limit	Upper Limit	
Apical	1 M	−0.09	−0.36	0.18	0.496
	1 Y	−1.27	−1.45	−1.1	<0.001 ***
	2 Y	−1.56	−1.77	−1.35	<0.001 ***
	Early activation	−3.46	−3.69	−3.22	<0.001 ***
	1 M: Early activation	3.12	2.8	3.44	<0.001 ***
	1 Y: Early activation	3.26	2.97	3.54	<0.001 ***
	2 Y: Early activation	3.17	2.88	3.45	<0.001 ***
Middle	1 M	−0.08	−0.27	0.1	0.389
	1 Y	−1.18	−1.35	−1.02	<0.001 ***
	2 Y	−1.34	−1.52	−1.16	<0.001 ***
	Early activation	−3.25	−3.41	−3.09	<0.001 ***
	1 M: Early activation	2.79	2.57	3.01	<0.001 ***
	1 Y: Early activation	3.06	2.82	3.29	<0.001 ***
	2 Y: Early activation	2.91	2.67	3.15	<0.001 ***
Basal	1 M	−0.09	−0.28	0.1	0.376
	1 Y	−0.58	−0.77	−0.39	<0.001 ***
	2 Y	−0.56	−0.78	−0.35	<0.001 ***
	Early activation	−3.35	−3.53	−3.17	<0.001 ***
	1 M: Early activation	2.98	2.73	3.24	<0.001 ***
	1 Y: Early activation	3.24	2.96	3.53	<0.001 ***
	2 Y: Early activation	3.15	2.84	3.47	<0.001 ***
Overall	1 M	−0.07	−0.26	0.11	0.454
	1 Y	−1.02	−1.17	−0.87	<0.001 ***
	2 Y	−1.16	−1.34	−0.99	<0.001 ***
	Early activation	−3.37	−3.54	−3.2	<0.001 ***
	1 M: Early activation	2.9	2.67	3.13	<0.001 ***
	1 Y: Early activation	3.2	2.97	3.44	<0.001 ***
	2 Y: Early activation	3.08	2.84	3.32	<0.001 ***

**Table 4:** Pairwise comparisons within each group for the apical, middle, basal, and overall electrode impedance between different time points. ns = non-significant, ** indicates *p* ≤ 0.01, *** indicates *p* ≤ 0.001, **** indicates *p* ≤ 0.0001.

Section	Study Group	Intervals Compared		Difference	Adjusted *p* Value	Significance
Apical	Classical activation	Pre-activation/1-Month	1-Year	1.25	<0.001	****
		Pre-activation/1-Month	2-Years	1.48	<0.001	****
		1-Year	2-Years	0.23	0.483	ns
	Early activation	Pre-activation	1-Month	−3.13	<0.001	****
		Pre-activation	1-Year	−1.86	<0.001	****
		Pre-activation	2-Years	−1.56	<0.001	****
		1-Month	1-Year	1.22	<0.001	****
		1-Month	2-Years	1.54	<0.001	****
		1-Year	2-Years	0.3	0.398	ns
Middle	Classical activation	Pre-activation/1-Month	1-Year	1.31	<0.001	****
		Pre-activation/1-Month	2-Years	1.45	<0.001	****
		1-Year	2-Years	0.15	0.510	ns
	Early activation	Pre-activation	1-Month	−2.77	<0.001	****
		Pre-activation	1-Year	−1.79	<0.001	****
		Pre-activation	2-Years	−1.53	<0.001	****
		1-Month	1-Year	0.92	<0.001	****
		1-Month	2-Years	1.19	<0.001	****
		1-Year	2-Years	0.26	0.044	**
Basal	Classical activation	Pre-activation/1-Month	1-Year	0.7	<0.001	****
		Pre-activation/1-Month	2-Years	0.76	<0.001	****
		1-Year	2-Years	0.05	1.0	ns
	Early activation	Pre-activation	1-Month	−3.02	<0.001	****
		Pre-activation	1-Year	−2.47	<0.001	****
		Pre-activation	2-Years	−2.43	<0.001	****
		1-Month	1-Year	0.47	0.002	***
		1-Month	2-Years	0.49	0.002	***
		1-Year	2-Years	0.04	1.0	ns
Overall	Classical activation	Pre-activation/1-Month	1-Year	1.09	<0.001	****
		Pre-activation/1-Month	2-Years	1.2	<0.001	****
		1-Year	2-Years	0.11	0.981	ns
	Early activation	Pre-activation	1-Month	−2.95	<0.001	****
		Pre-activation	1-Year	−2.09	<0.001	****
		Pre-activation	2-Years	−1.93	<0.001	****
		1-Month	1-Year	0.82	<0.001	****
		1-Month	2-Years	0.98	<0.001	****
		1-Year	2-Years	0.18	0.696	ns

## Data Availability

The datasets used and analyzed during the current study are available from the corresponding author upon reasonable request.
